# Clinical Performance of Implant-Supported Prostheses in the Rehabilitation of Patients Previously Treated for Medication-Related Osteonecrosis of the Jaws (MRONJ): A Systematic Review

**DOI:** 10.7759/cureus.61658

**Published:** 2024-06-04

**Authors:** Eduardo Anitua, Mohammad Alkhraisat, Asier Eguia

**Affiliations:** 1 Regenerative Medicine, Biotechnology Institute (BTI), Vitoria, ESP; 2 Research, Biotechnology Institute (BTI), Vitoria, ESP; 3 Estomatology II, University of The Basque Country (Universidad del País Vasco/Euskal Herriko Unibertsitatea), Leioa, ESP

**Keywords:** systematic review, implant-supported prosthesis, dental prosthesis, medication-related osteonecrosis of the jaw, dental implants

## Abstract

There is a lack of consensus on managing resultant bone and soft tissue defects or on restoring oral function and aesthetics following medication-related osteonecrosis of the jaws (MRONJ) lesion healing. This clinical challenge presents a dilemma for practitioners. Removable prostheses pose a recurrence risk if poorly fitted and may inadequately restore function or aesthetics in cases of significant bone defect. Dental implant-supported prostheses could enhance function and quality of life, though their risks and indications are not well-defined. This systematic review examines the clinical outcomes and complications associated with implant-supported rehabilitations post-MRONJ surgery. This study was conducted following the Preferred Reporting Items for Systematic Reviews and Meta-Analyses (PRISMA) statement recommendations and it was pre-registered in the Prospective Register of Systematic Reviews (PROSPERO) (CRD42023492539).

## Introduction and background

Introduction

MRONJ is a pathological condition characterized by the presence of necrotic (exposed or not) jawbone in the oral cavity, primarily associated with the prolonged use of antiresorptive, antiangiogenic, and other risk medications, wherein the mandible is more frequently affected than the maxilla [1-4]. More precisely, The American Association of Oral and Maxillofacial Surgeons (AAOMS) [1] defined MRONJ case as the presence of exposed bone or bone that can be probed through an intraoral or extraoral fistula(e) in the maxillofacial region that has persisted for more than eight weeks in a patient with current or past treatment involving antiresorptive agents, immune modulators, or antiangiogenic drugs. The presence of bone exposure or oral fistulae to comply with this case definition of MRONJ fails to include the so-called “stage 0” or non-exposed bone cases [5,6] No history of radiation therapy to the jaws or metastatic disease to the jaws is, in any case, required to fulfill MRONJ case definition [1]. MRONJ should be distinguished from other forms of oral and systemic osteonecrosis (ONJ) conditions [7-9] and identified by history and clinical examination [1].

Several studies have reported variable incidence rates reaching up to 15% in oncology patients receiving potent bisphosphonates (BPs) therapy [1,10]. There is a growing list of drugs associated with MRONJ with a variable level of evidence [3]. Most of them are employed in different medical fields such as endocrinology, orthopedics, or oncology to treat bone disorders caused by bone metastases, multiple myeloma, Paget disease, malignant hypercalcemia, or osteoporosis [1,3,11,12]. The duration of exposure, drug combinations, and different oral and systemic factors influence the risk for MRONJ [13,14]. Previous surgical procedures involving jaw bones, including dental extractions, periodontal surgery, or dental implant placement are also considered a relevant risk factor for MRONJ development [15-18]. Available data have shown a robust association between local infection [19] and periodontal disease [20] with MRONJ development. Nevertheless, there is no conclusive evidence or unanimous acceptance of the infection hypothesis [21,22].

Two decades after the first reports of MRONJ [23], the deep mechanisms underlying this condition's etiology and pathophysiology are still not fully understood and current nonoperative or operative treatment strategies are mostly empirical [4]. Ongoing research strives to unravel the intricate molecular mechanisms underlying MRONJ, paving the way for more precise diagnostics and tailored therapeutic interventions. Research has elucidated the complex interplay of factors contributing to MRONJ, including their influence on bone homeostasis, leading to compromised bone healing and increased susceptibility to necrosis and infection [12]. Among a long list of hypotheses [12,24], impaired angiogenesis is considered to play a pivotal role in the development of MRONJ by blocking angiogenesis via inhibition of cell proliferation [25,26]. Supporting this idea, reduced circulating growth factors have been measured in patients under bisphosphonate therapy [27,28]. Antiresorptive and antiangiogenic drugs also display cytotoxic effects in a dose and time-dependent manner on gingival fibroblasts, oral keratinocytes, and alveolar osteoblasts, causing a decrease in cell proliferation, viability, and migration [12,29].

The diagnosis and classifications of MRONJ severity have evolved [30,31], and currently, various radiographic imaging modalities play a crucial role in the diagnostic algorithm for MRONJ [32,33]. CBCT, CT, MRI, and nuclear medicine provide valuable insights into the extent of bone involvement, the presence of sequestra, and soft tissue abnormalities [32-34]. Biopsy remains the gold standard for confirming MRONJ diagnosis [35]. Histopathological analysis of biopsy specimens aids in distinguishing MRONJ from other conditions, such as osteomyelitis or malignancy [36].

Preventive measures and guidelines for patient management are continually refined based on emerging evidence [1]. Clinical approaches to treat MRONJ involve a multidisciplinary strategy, including discontinuation or modification of the causative medication (if possible), antimicrobial therapy to manage infections, and surgical interventions such as debridement or resection of necrotic bone [37-39]. Complex surgical approaches are frequently required for more severe cases [40]. Platelet-rich plasma concentrates (PRPs) [41,42] and recombinant human parathyroid hormone (Teriparatide) [43] are gaining attention as an adjunctive treatment in MRONJ management and prevention, by enhancing angiogenesis and osteoanabolic properties, among other effects.

Once the healing of a MRONJ lesion has been achieved, there is no clear consensus on how to deal with the bone and soft tissue defects secondary to the treatment or how to restore patients´ oral function and aesthetics. A dilemma arises for the clinician when facing this clinical situation. In certain scenarios, removable prostheses may prove insufficient for the complete restoration of oral function and aesthetics, while poor fitting also exerts a risk factor for recurrence. Dental implant-supported prosthesis may be an option to restore function and quality of life for these patients, but possible risks and indications have not been clearly elucidated. This systematic review explores the available information related to the clinical performance and complications of implant-supported rehabilitations performed after MRONJ surgical treatment.

## Review

Materials and methods

A systematic review was conducted in accordance with the Preferred Reporting Items for Systematic Reviews and Meta-Analyses (PRISMA) guidelines [44,45] to address the following PIO question: In patients previously treated for (antiresorptive) MRONJ (P), What is the clinical performance (O) of implant-supported rehabilitation (I)? This review aimed to provide clinicians with the best available evidence to guide the rehabilitation of patients post-MRONJ treatment.

Protocol and registration

The review was registered in the International Prospective Register of Systematic Reviews (PROSPERO) of the National Institute for Health Research (NIHR) (CRD42023492539) before the review onset. The PRISMA guidelines [44,45] were followed throughout the review process.

Eligibility criteria, Information sources, and search strategy

The following electronic databases were searched: Medline (PubMed), Scopus, and EBSCO. The search strategy (PIO) was formulated employing the following considerations: Patient (MRONJ surgically treated Patients); Intervention (Implant-supported rehabilitation after MRONJ treatment); Outcomes (Implant survival, marginal bone level, technical complications, disease recurrence and patient management).

The main question built was then as follows: “In patients previously treated for (Antiresorptive) MRONJ (P), What is the clinical performance (O) of implant-supported rehabilitation (I)?”. In the search strategy, the following terms were employed: “dental implants” (MeSH Term), “MRONJ” (free term), “reconstruction” (free term), and rehabilitation” (MeSH Term) (Table 1).

**Table 1:** Employed search query terms

Search query
("MRONJ"[All Fields] AND ("plastic surgery procedures"[MeSH Terms] OR ("plastic"[All Fields] AND "surgery"[All Fields] AND "procedures"[All Fields]) OR "plastic surgery procedures"[All Fields] OR "reconstruction"[All Fields] OR "reconstructions"[All Fields] OR "reconstruct"[All Fields] OR "reconstructability"[All Fields] OR "reconstructable"[All Fields] OR "reconstructed"[All Fields] OR "reconstructible"[All Fields] OR "reconstructing"[All Fields] OR "reconstructional"[All Fields] OR "reconstructive"[All Fields] OR "reconstructs"[All Fields])) OR ("MRONJ"[All Fields] AND ("rehabilitant"[All Fields] OR "rehabilitants"[All Fields] OR "rehabilitate"[All Fields] OR "rehabilitated"[All Fields] OR "rehabilitates"[All Fields] OR "rehabilitating"[All Fields] OR "rehabilitation"[MeSH Terms] OR "rehabilitation"[All Fields] OR "rehabilitations"[All Fields] OR "rehabilitative"[All Fields] OR "rehabilitation"[MeSH Subheading] OR "rehabilitation s"[All Fields] OR "rehabilitational"[All Fields] OR "rehabilitator"[All Fields] OR "rehabilitators"[All Fields])) OR ("MRONJ"[All Fields] AND ("dental implants"[MeSH Terms] OR ("dental"[All Fields] AND "implants"[All Fields]) OR "dental implants"[All Fields]))

This search was additionally complemented by: a complementary manual search in the same databases (including other terms such as “BRONJ”, “osteonecrosis of the jaws”, “prosthetic”, “healed” or “healing”), a review of the reference lists of the full-text selected articles, manual searching in additional databases (DOAJ, University of London Online Library), Grey literature searching (WorldCat, WorldWideScience, Open Access Theses and Dissertations) and Internet free search using the terms “dental implants”, “MRONJ”, “reconstruction” and “rehabilitation”.

Articles in all languages published between January 2003 (the year of the first descriptions of MRONJ in the literature) and December 2023 were initially selected. The title and abstract of the publications were assessed by two authors independently. The inclusion for the studies were: Patients previously treated for MRONJ and Patients subsequently rehabilitated with an implant-supported prosthesis. The following were considered as exclusion criteria: Review, survey, or consensus reports; No placement of dental implants and In vitro or preclinical research.

Study selection and data collection process

The same two independent reviewers performed the study selection. In case of disagreement a third reviewer acted. Article selection was based on the abstract and the article selection criteria. Both reviewers determined whether the selected articles finally met the inclusion criteria for this review after reading the complete articles. Cohen’s kappa coefficient, with a κ value of 0,89. (98.34% of agreement) was used to assess the agreement in the selection process.

Both researchers independently collected data in duplicate from all articles and then pooled them in the same worksheet. From each selected study the following information was extracted: Authors, type of study, year of publication, number of patients, gender, age (or mean age in case series when not detailed; Type of antiresorptive therapy, MRONJ location, MRONJ treatment; Number of implants placed, implant location, healing time before implant placement, preoperative and postoperative medication, antiresorptive therapy continuation/discontinuation, type of implant surgery, type of implants, simultaneous bone grafting data, implant size, time to implant loading, type of prosthetic restoration; Follow-up period (or mean follow-up period when not detailed), implant survival, technical complications, disease (MRONJ) relapse, peri-implant marginal bone level and any other postoperative complications.

Data Synthesis and Outcomes

After data extraction, the main outcomes analyzed were: Implant survival (defined as the presence of the implant in function in the mouth after the end of the follow-up period); Complications (including technical complications affecting both the implant or the prosthesis and all types of biological complication affecting the bone or soft tissues); Peri-implant marginal bone stability (measured through Rx follow-up); Disease (MRONJ) recurrence (new bone lesions related or not to dental implant position); and Patient management (MRONJ treatment and pre-implant surgery medication).

Risk of Bias in Individual Studies

To assess the methodological quality of the included articles, the “NIH quality assessment tools'' for case series and for observational cohort and cross-sectional studies were employed. Although originally designed to aid reviewers, these tools have become widely employed in numerous recent systematic reviews for evaluating study quality [46,47]. Joanna Briggs Institute (JBI) critical appraisal checklist was used to assess case reports [48]. Scores higher than 70% were classified as having a high quality (Good), those with a score between 50% and 70% as having a medium quality (Fair), and those with a score less than 50% as having a low quality (Poor). The risk of bias was independently assessed by two authors, with any discrepancies resolved through the involvement of a third author.

The Overall Risk of Bias

GRADE (Grading of Recommendations, Assessment, Development and Evaluations) system was employed for the collective assessment of the risk of bias across all included studies [49]. The quality of evidence was assessed in each individual study based on five factors: risk of bias (flaws in study design or execution), inconsistency (variability in results across studies), indirectness (evidence not directly applicable to the population, intervention, or outcomes of interest), imprecision (wide confidence intervals or small sample sizes), and publication bias (evidence of selective publication). After evaluating these factors, the quality of evidence was rated as high (very confident in the effect estimate), moderate (moderately confident, with some possibility of a substantial difference), low (limited confidence, with a substantial difference likely), or very low (very little confidence).

Summary Measures

All variables were gathered into a database and analyzed using IBM SPSS Statistics v. 20.0 (IBM Corp., Armonk, NY, USA). Basic descriptive statistics were used for univariate description.

Results

Study Selection

**Figure 1:**
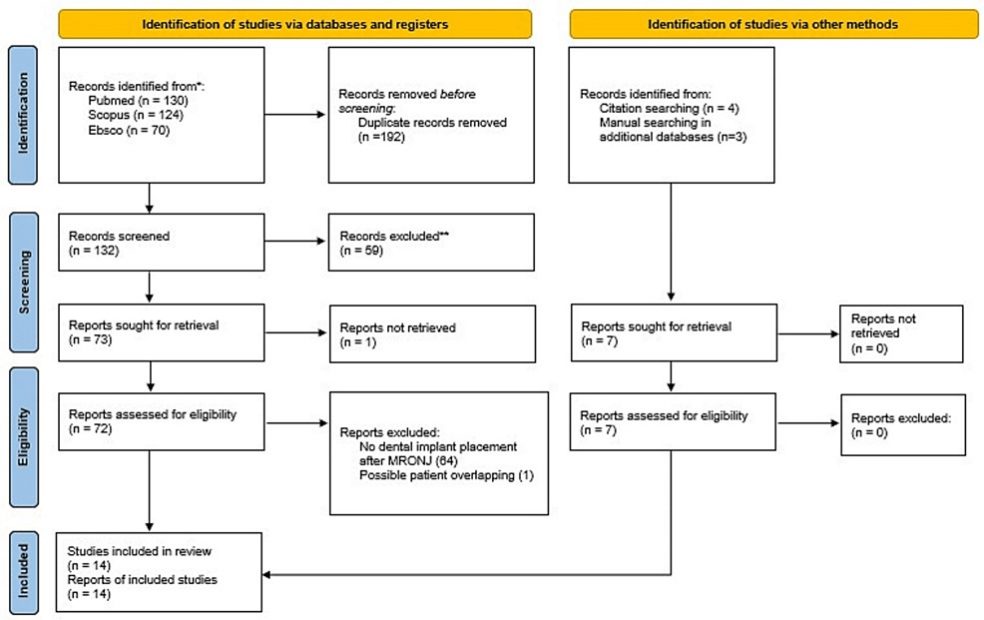
Study selection workflow Study selection workflow diagram following Preferred Reporting Items for Systematic Reviews and Meta-Analyses (PRISMA) guidelines.

The initial search allowed to identify 324 articles and additional searches uncovered seven more articles. Before Screening, 192 duplicate articles were removed, and 59 other articles were also removed after the title/abstract review. A total of 73 articles were targeted for retrieval, out of which 72 were evaluated for eligibility. After a deep analysis of the article, 64 were excluded as no dental implants were placed after MRONJ placement. Another article [50] was excluded to prevent patients from overlapping with another already included study with patients from the same series [51]. Longer follow-up studies were prioritized. Data from subgroup 1 of the study of Hjortholt et al. [51] were excluded as these patients did not suffer MRONJ. Only data from patients who received dental implants were retrieved from the retrospective study from Caldroney et al. [52] and from the case series from Kasper et al. [53]. Finally, 14 articles were included in the review [51-64]. A summary of the study selection process in a Flow Diagram adapted from Page et al. [45] is presented in Figure 1.

Study Characteristics

The 14 articles finally included in the review [51-64] corresponded to 12 case reports/series, one prospective observational study, and one retrospective study that involved 25 patients and 52 implants. All the included articles were published from 2008 onwards. No previous systematic reviews or randomized controlled trials (RCTs) were identified during the literature search.

Risk of Bias Within Studies

Most of the included articles were case reports/series and nearly half of the patient-based pooled information (48%) came from this type of study which is often associated with certain methodological limitations such as selection bias (as typically involves the description of a single or a small number of cases), lack of comparison groups and lack of generalizability. The study from Hjortholt et al. [51] (prospective observational study) included 40% of the included patients in this review.

Synthesis of Results

The 14 finally selected studies provided data from 25 patients (13 females/two males/10 not disclosed) with a mean age of 68 years and 52 implants placed after MRONJ treatment. Antiresorptive therapy with BPs or denosumab was stated in all cases, with the previous history of MRONJ much more frequently reported in the mandible (Table 2).

**Table 2:** Selected articles details (I) Selected articles, year of publication, type of study, and medication-related osteonecrosis of the jaws (MRONJ) history data.

Authors	Study type	Antiresorptive therapy	MRONJ location	MRONJ treatment
Goker et al., 2023 [54]	Case report	Alendronate	Right maxillary region	Resection surgery
Rahim et al., 2024 [55]	Case report	Alendronate	Right body of the mandible	Partial mandibulectomy with titanium reconstruction plate
Hjortholt et al., 2023 [51]	Prospective (observational) study	Pamidronate, Zoledronate, Alendronate, Ibandronate or Denosumab	NA	Block resection
Lee et al., 2023 [56]	Case report	Alendronate	Right posterior mandible	Sequestrectomy, saucerization, and removal of the 2 involved implants
Caldroney et al., 2017 [52]	Retrospective study	Pamidronate and/or Alendronate	(Symphysis/left mandible) and (mandibular body including symphysis)	Reconstruction with fibula
Ottesen et al., 2022 [57]	Case report	Denosumab	Nonexposed MRONJ bilaterally in the mandible	Surgical removal
Kasper et al., 2023 [53]	Case report	Zoledronate	Left premolar region	Partial mandible resection with free fibula flap
Ferrari et al., 2008 [58]	Case report	Pamidronate and zoledronate	Entire mandible	Total mandibulectomy with a fibula free flap after.
Rugani et al., 2015 [59]	Case report	Ibandronate	Left lower third molar region	Removal of necrotic parts of the jawbone
Kim et al., 2016 [60]	Case report	Alendronate	(left upper canine and first premolar area) and (left upper first molar and second premolar area)	Sequestrectomy and implant/tooth removal
Ahn et al., 2014 [61]	Case report	Alendronate	Right mandibular body and the symphysis	Marginal mandibulectomy with metal plates
Kim et al., 2019 [62]	Case report	Uncertain type/dosage biphosphonate history	Anterior mandible bilaterally	Sequestrectomy including debridement and implant removal
Anitua E, 2017 [63]	Case report	Zoledronate	Right posterior mandible	Resection of necrotic bone and pure PRP application.
Teramoto et al., 2018 [64]	Case report	Zoledronate	Anterior and posterior mandible	Segmental resection and fibula flap

MRONJ treatments ranged from sequestrectomy to block resection and free fibula flap reconstruction and the healing time to implant placement ranged from 0 to 31 months. Among the patients with healing time information available, 84% of implants were placed within the first year after treatment, and 16% more than one year after MRONJ treatment (Table 3).

**Table 3:** Selected articles details (II) Selected articles, demographic data, the healing time before implant placement, and medication. * Does not disclosed for the patients at dental implant placement.

Authors	Number of patients	Patient age (years)	Patient sex	Healing time before implant placement (months)	Preoperative and postoperative medication	Discontinue antiresorptive therapy
Goker et al., 2023 [54]	1	78	Female	8	1 g of augmentin for 7 days starting one day before surgery	Yes
Rahim et al., 2024 [55]	1	71	Female	19	NA	Yes
Hjortholt et al., 2023 [51]	4	Mean: 64 (range: 40-78)	NA*	NA	Two days preoperatively and 12 days postoperatively with 500 mg amoxicillin and 125 mg clavulanic acid tablets (thrice daily). If penicillin allergy, 300 mg clindamycin capsules (thrice daily)	No
	6	Mean: 74 (range: 57-88)	NA*	0		
Lee et al., 2023 [56]	1	80	Female	11	One week before and one week after surgery. amoxicillin–clavulanic acid and zaltoprofen	No (switch to non-BP therapy using raloxifene)
Caldroney et al., 2017 [52]	2	60 and 61	Female	NA	NA	NA
Ottesen et al., 2022 [57]	1	71	Male	12	Implant surgery: two days preoperatively and 9 days postoperatively. 500 mg amoxicillin and 125 mg clavulanic acid (three times daily). Abutment surgery: one‐shot prophylaxis with 1000 mg amoxicillin and 250 mg clavulanic	Yes
Kasper et al., 2023 [53]	1	69	Female	12	NA	NA
Ferrari et al., 2008 [58]	1	66	Male	0	Postoperatively, topical and systemic antibiotic therapy with meropenem and gentamicin for one month	Yes
Rugani et al., 2015 [59]	2	64	Female	9	NA	Yes
Kim et al., 2016 [60]	1	73	Female	17	Prophylaxis amoxicillin with clavulanic acid for seven days	Yes
Ahn et al., 2014 [61]	1	63	Female	9	NA	Yes
Kim et al., 2019 [62]	1	Late 60s	Female	9	High-concentration antibiotics (not further specified)	Yes
Anitua E, 2017 [63]	1	50	Female	31	NA	Yes
Teramoto et al., 2018 [64]	1	73	Female	10	NA	NA

Antiresorptive therapy was not discontinued in 52.4% of patients and was not disclosed in 4/25. Only 2/52 implants (from the same patient) corresponded to zygomatic implants, the rest screw implants (Table 3) supporting fixed rehabilitations (29/48; 60%), removable prostheses (19/48; 40%) and not disclosed in 4/52.

Immediate loading with fixed partial prosthesis was employed to restore only one patient, and in the rest, submerged healing was preferred (except for another patient with a one-stage approach) with a time until implant loading that ranged from 3 to 12 months (Table 4).

**Table 4:** Selected articles details (III) Selected articles, implant treatment-related retrieved data. * Within parenthesis indicates the number of implants. # ePTFE: expanded polytetrafluoroethylene.

Authors	Number of implants	Implant type	Implant surgery	Simultaneous Bone grafting	Implant length (mm)*	Implant diameter (mm)*	Location	Time to load (months)	Prosthesis type*
Goker et al., 2023 [54]	2	Zygomatic	Submerged healing	No	45 and 35	3.5/4.2	Posterior maxilla	6	Screw-retained Toronto prosthesis
Rahim et al., 2024 [55]	1	screw	Submerged healing	No	9.5	3.5	Posterior mandible	8	Partial lower cobalt chrome overdenture
Hjortholt et al., 2023 [51]	6	screw	Submerged healing	No	NA	NA	Maxilla (1), Mandible (5)	≥ 3	Crown (6)
	16	screw			NA	NA	Maxilla (3), Mandible (13)		Crown (1); Removable dental prosthesis (5)
Lee et al., 2023 [56]	3	screw	Submerged healing	Autogenous bone and xenograft covered with ePTFE^#^ membrane	10 (1), 15 (2)	4 (2), 5 (1)	Right mandibular first premolar, second premolar, and second molar	4	Fixed partial prosthesis
Caldroney et al., 2017 [52]	NA	NA	NA	NA	NA	NA	NA	NA	NA
Ottesen et al., 2022 [57]	4	screw	Submerged healing	No	NA	NA	Second premolars and canines	8	Fixed partial prosthesis with cantilever extension (2)
Kasper et al., 2023 [53]	4	screw	NA	No	NA	NA	Mandible	NA	NA
Ferrari et al., 2008 [58]	6	screw	Submerged healing	No	13	3.5 (1) and 4.3 (5)	Mandible	12	Toronto Brånemark prosthesis
Rugani et al., 2015 [59]	2	screw	Submerged healing	No	9.5 and 11	5.5 and 4.5	Lower left first and second molars	4	Crown (2)
Kim et al., 2016 [60]	3	screw	Submerged healing	No	10	4	left upper lateral incisor, canine, and first premolar	5	Fixed partial prosthesis
Ahn et al., 2014 [61]	2	screw	Submerged healing	No	NA	NA	Left lower canine and right lower canine	4	Complete overdenture
Kim et al., 2019 [62]	1	screw	One-stage approach	No	10	4	Left lower can ine	NA	Magnetic attachment overdenture
Anitua E, 2017 [63]	2	screw	Immediate loaded	No	NA	NA	Right lower second premolar and second molar	0	Fixed partial prosthesis
Teramoto et al., 2018 [64]	5	Screw	Submerged healing	No	9.5	4.5	Anterior mandible (3), posterior mandible (2)	16	Removable and fixed prosthesis.

Follow-up periods longer than two years were only reported in three studies (three patients). The marginal bone level was scarcely reported (Table 5), and Implant survival was 50/52 (96.1%). New bone-exposed lesions were reported in three patients, being related to dental implant position only in one case. The information about technical complications was not disclosed except in one study [51] including 10/52 patients (one patient; screw loosening after one year).

**Table 5:** Selected article details (IV) Selected articles; follow-up time and outcomes after implant placement.

Authors	Follow-up time of the dental implants (years)	Survival	Marginal bone loss	Newly exposed bone lesion	Related to dental implant position	Position	Prognosis and management	Technical complications
Goker et al., 2023 [54]	3	All	NA	No	-	-	-	NA
Rahim et al., 2024 [55]	4	All	NA	No	-	-	-	NA
Hjortholt et al., 2023 [51]	0,5 to 2	All	1,75 mm (1 implant), all other implants ≤ 1,0 mm	yes (2)	No	Lingually-posterior mandible	Both patients had a progression of their cancer. One of the patients died shortly after the 1 year follow-up visit, the other patient was treated successfully non-surgically after a control regimen. The exposed bone sequestered spontaneously after 2 months, and no recurrences occurred.	Screw loosening after 12 months (1)
	< 0,5 to 2	All	All ≤ 1,0 mm					None
Lee et al., 2023 [56]	7	All	NA	No	-	-	-	NA
Caldroney et al., 2017 [52]	NA	All	NA	No	-	-	-	NA
Ottesen et al., 2022 [57]	1	2	Periimplantitis or MRONJ around 2 implants	Yes	Yes	Around the implants in the implants at 23 and 24	Secuestrum and dental implant removal	None
Kasper et al., 2023 [53]	NA	All	NA	NA	NA	NA	NA	NA
Ferrari et al., 2008 [58]	2	All	NA	No	-	-	-	NA
Rugani et al., 2015 [59]	1.3	All	NA	No	-	-	-	NA
Kim et al., 2016 [60]	1.5	All	NA	No	-	-	-	NA
Ahn et al., 2014 [61]	1.9	All	NA	No	-	-	-	NA
Kim et al., 2019 [62]	2	All	NA	No	-	-	-	NA
Anitua E, 2017 [63]	1	All	NA	No	-	-	-	-
Teramoto et al., 2018 [64]	NA	All	NA	No	-	-	-	-

Risk of Bias Across Studies

To assess each individual study's quality, the following tools were employed: the “NIH - Study Quality Assessment Tool for Observational Cohort and Cross-sectional Studies” for prospective or retrospective observational cohort studies and the “Joanna Briggs Institute (JBI) critical appraisal checklist” to assess case reports. Nine articles were rated as “Fair”, 3 as “Poor” and 2 as “Good” (Tables 6, 7). As a result of the type of study design in selected studies and the great heterogeneity found in methodological aspects, a quantitative analysis followed by meta-analysis was not possible.

**Table 6:** Quality assessment of included articles I Quality assessment of included articles: Cohort studies: (1) Was the research question or objective in this paper clearly stated? (2) Was the study population clearly specified and defined? (3) Was the participation rate of eligible persons at least 50%? (4) Were all the subjects selected or recruited from the same or similar populations (including the same time period)? Were inclusion and exclusion criteria for being in the study prespecified and applied uniformly to all participants? (5) Was a sample size justification, power description, or variance and effect estimates provided? (6) For the analyses in this paper, were the exposure(s) of interest measured prior to the outcome(s) being measured? (7) Was the timeframe sufficient so that one could reasonably expect to see an association between exposure and outcome if it existed? (8) For exposures that can vary in amount or level, did the study examine different levels of the exposure as related to the outcome (e.g., categories of exposure, or exposure measured as a continuous variable)? (9) Were the exposure measures (independent variables) clearly defined, valid, reliable, and implemented consistently across all study participants? (10) Was the exposure(s) assessed more than once over time? (11) Were the outcome measures (dependent variables) clearly defined, valid, reliable, and implemented consistently across all study participants? (12) Were the outcome assessors blinded to the exposure status of participants? (13) Was the loss to follow-up after baseline 20% or less? (14) Were key potential confounding variables measured and adjusted statistically for their impact on the relationship between exposure(s) and outcome(s)? * Yes; -  No; o N.A.: not applicable/N.R.: not disclosed. NIH: National Institute of Health

NIH Quality Assessment Tool for Observational Cohort and Cross-Sectional Studies																
Authors	Study type	1	2	3	4	5	6	7	8	9	10	11	12	13	14	Rating
Hjortholt et al., 2023 [51]	Prospective Observational Study	*	*	o	*	-	*	*	*	*	*	*	o	o	-	FAIR
Caldroney et al., 2017 [52]	Retrospective (single center) Study	*	*	*	*	-	o	*	*	*	-	-	o	o	-	FAIR

**Table 7:** Quality assessment of included articles (II) Quality assessment of included articles: Case reports: (1) Were the patient’s demographic characteristics clearly described? (2) Was the patient’s history clearly described and presented as a timeline? (3) Was the current clinical condition of the patient on presentation clearly described? (4) Were diagnostic tests or assessment methods and the results clearly described? (5) Was the intervention(s) or treatment procedure(s) clearly described? (6) Was the post-intervention clinical condition clearly described? (7) Were adverse events (harms) or unanticipated events identified and described? (8) Does the case report provide takeaway lessons? * Yes; -  No; o N.A.: not applicable / N.R.: not disclosed. JBI: Joanna Briggs Institute.

JBI Critical appraisal checklist for case reports										
Author	Study type	1	2	3	4	5	6	7	8	Rating
Goker et al., 2023 [54]	Case report	*	*	*	-	*	-	o	-	FAIR
Rahim et al., 2024 [55]	Case report	*	*	-	-	-	-	o	-	POOR
Ottesen et al., 2022 [57]	Case report	*	*	*	-	*	-	o	-	FAIR
Kasper et al., 2023 [53]	Case report	*	*	*	-	*	*	*	*	GOOD
Ferrari et al., 2008 [58]	Case report	*	*	*	-	*	*	o	*	GOOD
Rugani et al., 2015 [59]	Case report	*	*	-	-	*	-	o	-	POOR
Kim et al., 2016 [60]	Case report	*	*	*	*	-	-	o	-	FAIR
Ahn et al., 2014 [61]	Case report	*	*	*	-	*	-	o	-	FAIR
Kim et al., 2019 [62]	Case report	*	*	*	-	*	-	o	-	FAIR
Anitua E, 2017 [63]	Case report	-	*	*	*	*	-	o	-	FAIR
Teramoto et al., 2018 [64]	Case report	-	*	*	*	-	-	o	*	FAIR
Ottesen et al., 2022 [57]	Case report	*	*	*	-	-	-	o	-	POOR

Strength of Evidence (SoE)

As no randomized studies were identified, the level of evidence was initially rated as “Low”, attending GRADE (Grading of Recommendations, Assessment, Development and Evaluations) system [49]. Further assessment of other domains that could rate up (Large magnitude of effect, Dose-Response gradient, Confounding factors) or down (Risk of bias, Imprecision, Inconsistency, Indirectness, and Publication bias), the SoE evaluation was downrated to “Very Low”.

Discussion

In the short follow-up time, implant-supported rehabilitation in patients with a history of previous MRONJ surgical treatment presented a low rate of biological complications, reduced incidence of disease recurrence (in relation to implants), and acceptable implant survival, with a “very low” strength of evidence. The heterogeneity in surgical treatments for MRONJ and pre-implant surgery medication regimens precluded the derivation of clinical recommendations. The considerable heterogeneity in the locations (although mostly were in the mandible) and staging of MRONJ lesions likely correlated with the variability in performed treatments and pre- and post-surgical medication and care.

The postoperative rehabilitation of edentulous space after the treatment of the MRONJ lesion is challenging. The utilization of removable prostheses, in many instances, proves inadequate due to insufficient retention and the potential for ill-fitting dentures to compromise tissue stability [65,66]. In patients with a history of MRONJ, there is still no clear consensus or guidelines on how to deal with the bone and soft tissue defects secondary to the surgical approach treatment or how to ideally restore patients’ oral function and aesthetics. Furthermore, it has not been definitively established whether patients with a previously healed MRONJ are at higher risk than other patients at risk of developing a secondary MRONJ lesion after implant-supported rehabilitation [13,16,18,37,40]. Regardless, several considerations should be considered when contemplating the rehabilitation with an implant-supported prosthesis in each individual patient after MRONJ treatment. As in other patients-at-risk, [66] the management of these patients requires a comprehensive approach to minimize the risk of exacerbating the disease or triggering a new MRONJ. Among them, medical history re-assessment (and periodic updating) is strictly necessary. Patient's medical history, with specific attention to the type, duration, and dosage of antiresorptive or antiangiogenic medications, as well as the presence of any other comorbidities should be thoroughly evaluated [1,65]. Possible synergistic effects [25] between drugs should be further assessed to establish the risk level as multiple-drugs MRONJ is occasionally related to disease onset or worsening [66]. Effective communication with the patient's primary healthcare provider ensures a comprehensive understanding of the patient's medical condition and enhances collaborative decision-making. It can also be relevant for mandatory risk stratification and for the (controversial) decision about the cessation of risk medications, which should not be decided by the dentist on his own [65,67]. In patients with previous MRONJ, detailed preoperative imaging evaluation, multidisciplinary approach, the use of meticulous surgical techniques to minimize trauma to oral tissues, tailored antibiotic prophylaxis, postoperative monitoring, patient education, or informed consent recommendations should not reasonably differ from other patients-at-risk [1,68-70].

Antiresorptive medications (especially intravenous BPs) have been related to reduced implant survival and impaired osseointegration [71-72] or increased marginal bone loss (MBL) [73]. However, the risk of implant failure in intraorally treated osteoporotic patients seems not to be increased [74,75]. Failed implants in bisphosphonate patients do not necessarily lead to osteonecrosis [71].

In the studies included in this review, favorable implant survival could be observed in the short follow-up (mostly information came from studies with less than two-year follow-up), but lack of data made it unable to clearly assess MBL. It was also not possible to ascertain differences between implants placed in the pristine bone of the patient or in grafted bone (fibula flap grafting), which could be helpful in future research to further elucidate the pathophysiologic aspects of MRONJ. It could not be conclusively determined whether a fixed or a removable prosthetic solution is superior. Literature evidence suggests that wearing ill-fitting dentures is a risk factor for developing MRONJ [65,76]. Therefore, in cases where a removable solution has been preferred, a strict clinical follow-up is highly advisable to readjust the prostheses when needed, to avoid soft tissue injuries.

Antiresorptive therapy was discontinued in most of the cases included in this review, although cessation of antiresorptive agents is controversial, and there is limited evidence that cessation contributes to preventing MRONJ development [65]. The half-life of BPs remains unclear (hypothesized to be 10 years) as they bind to bone matrix [1,65]. However, there is probably a large individual variability in the rate of bone remodeling (related to genetic and systemic conditions), that could influence the precise bone effect lasting of BPs in each individual patient [77]. The half-life of denosumab has been better clarified and claimed to last for 26 days after administration [78,79]. Nevertheless, denosumab bone effects continue for longer times [78-80]. Ideal timing for implant-supported rehabilitation after MRONJ treatment remains insufficiently clarified.

The nature and quality rating of the included articles are remarkable study limitations in this review. Case reports could lead to publication bias as there is a tendency to publish cases with unusual treatments, results, or dramatic findings. This publication bias could lead to an overrepresentation of cases with unrepresentative outcomes, creating a skewed perception of the prevalence and significance of certain conditions or interventions. The number of included patients and follow-up time may be other noteworthy limitations that need to be considered when analyzing the findings of this study.

## Conclusions

Implant-supported rehabilitation success after heterogeneous MRONJ surgical treatments has been described despite limited empirical support. It is of paramount relevance that these challenging cases be multidisciplinary treated under controlled conditions, by experienced clinicians and in compliance with preventive measures and surgical considerations.

The very low strength of evidence rating precluded the formulation of definitive clinical recommendations. New high-evidence, low-bias research is needed. Particularly, reporting both positive and negative clinical findings in similar scenarios will help reduce publication bias and expand the evidence base for clinical decision-making.

## Appendices

**Table 8:** Terms and search strategies adapted for each database.

Database	Terms and search strategy
Medline	((MRONJ AND reconstruction) OR (MRONJ AND rehabilitation)) OR (MRONJ AND dental implants)
Scopus	TITLE-ABS-KEY (MRONJ AND rehabilitation) OR TITLE-ABS-KEY (MRONJ AND reconstruction) OR TITLE-ABS-KEY (MRONJ AND dental AND implants)
EBSCO	((MRONJ AND reconstruction) OR (MRONJ AND rehabilitation)) OR (MRONJ AND dental implants)
